# From starch to bioactives: emerging trends in taro (*Colocasia esculenta* L.) research on composition, functionality, health benefits, and sustainable food potential

**DOI:** 10.3389/fnut.2025.1640156

**Published:** 2025-09-09

**Authors:** Yuan Tan, Feng-Jin Zheng, Bo Lin, Jing Chen, Krishan K. Verma, Gan-Lin Chen

**Affiliations:** ^1^College of Agriculture, Guangxi University, Nanning, Guangxi, China; ^2^Institute of Agro-Products Processing Science and Technology, Guangxi Academy of Agricultural Sciences, Nanning, Guangxi, China; ^3^Guangxi Subtropical Crops Research Institute, Guangxi Academy of Agricultural Sciences, Nanning, Guangxi, China; ^4^Key Laboratory of Quality and Safety Control for Subtropical Fruit and Vegetable, Ministry of Agriculture and Rural Affairs, Nanning, China; ^5^Guangxi Key Laboratory of Quality and Safety Control for Subtropical Fruits, Nanning, Guangxi, China; ^6^Sugarcane Research Institute, Guangxi Academy of Agricultural Sciences, Nanning, Guangxi, China

**Keywords:** anti-nutritional factors, antimicrobial activity, anti-obesity potential, functional foods, prebiotic potential, *Colocasia esculenta* L.

## Abstract

Taro (*Colocasia esculenta* L.), a globally significant root crop, has garnered renewed scientific interest due to its nutritional richness, bioactive compounds, and diverse functional applications. Recent studies have elucidated its unique composition, including high-quality starch with low glycemic potential, dietary fiber, polyphenols, i.e., flavonoids, anthocyanins, and potassium, calcium, iron minerals. Innovations in processing strategies, such as fermentation and thermal applications, have enhanced its digestibility while mitigating anti-nutritional factors like oxalates. Functionally, taro exhibits prebiotic, hypoglycemic, and hypocholesterolemic properties, attributed to its resistant starch and antioxidant activity. Taro corms starch (70%−80% on dry basis) contemplate as a cheapest abode for food industries due to its multiple functions in foods, such as stabilizer, emulsifier, fat substitute, and as filler agent too. It is rich in mucilage and starch granules, making it a highly digestible (99%) ingredient because of their small in size. Starch is a complex carbohydrate synthesized in some plant species, i.e., rice, wheat, potato, taro, elephant foot yam, maize, and others. Taro starches have higher phosphorus (0.407%), protein (5.605%) and ash (0.851%) contents than other tropical roots like tiger nut and sweet potato, but lower lipid content (0.283%). Taro has been found to contain several active compounds, such as resistant starch, mucilage, anthocyanins, hemagglutinin, non-starch polysaccharides, protein, tarin lectin, and others, which exhibit numerous beneficial properties, including antitumor, antimetastatic, antioxidant, and anti-inflammatory effects. Emerging evidence highlights its efficacy in modulating gut microbiota, reducing inflammation, and mitigating risks of metabolic disorders, such as diabetes and obesity. Furthermore, taro-derived bioactive compounds show promise in antimicrobial and anticancer applications. Advances in genomics and biofortification are driving sustainable cultivation and novel food approaches, including gluten-free products and functional food additives. Despite significant advancements, challenges remain in standardizing and scaling up these bioactive extraction processes for industrial applications. This review emphasizes taro's potential as a vital crop for food security and human health due to recent research advancements.

## Graphical abstract

**Figure:**
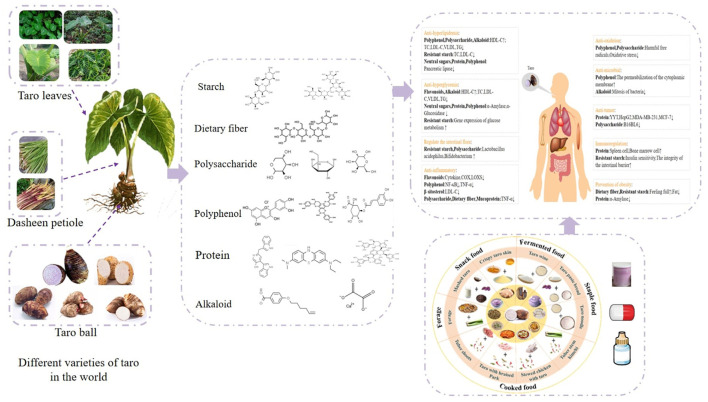

## 1 Introduction

**Figure 1:**
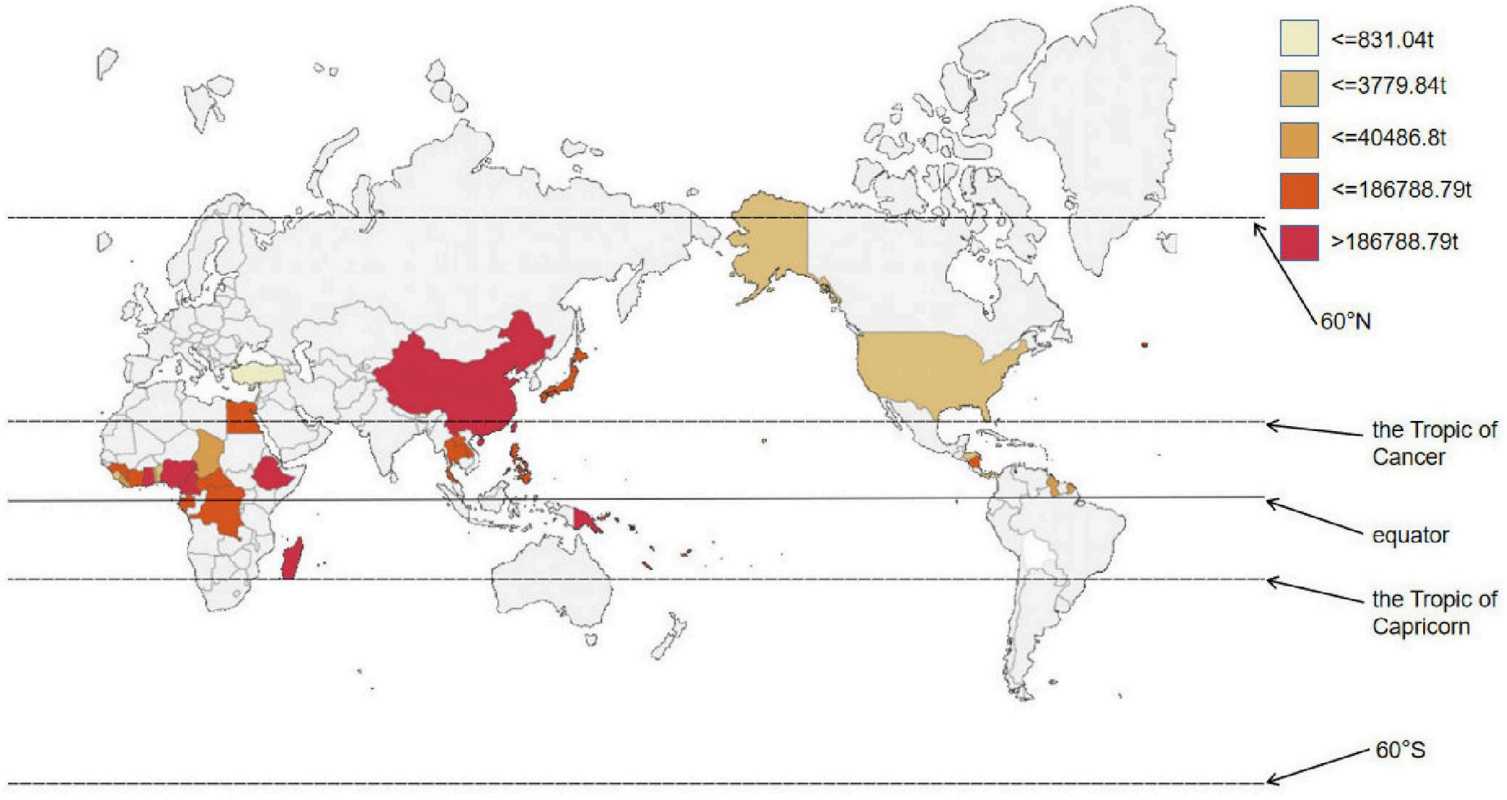
Global distribution of taro production.

**Figure 2:**
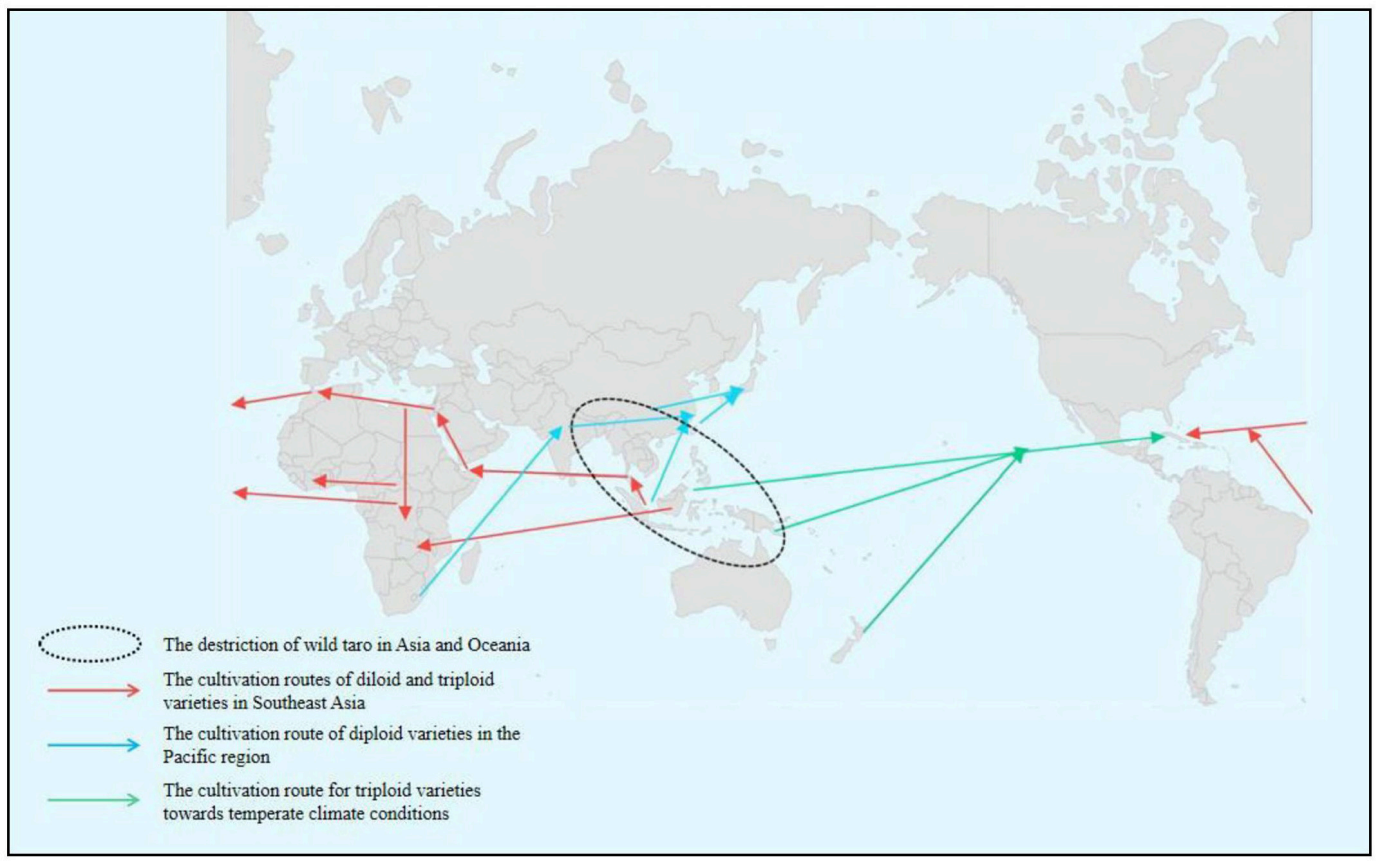
World taro migration map.

**Figure 3:**
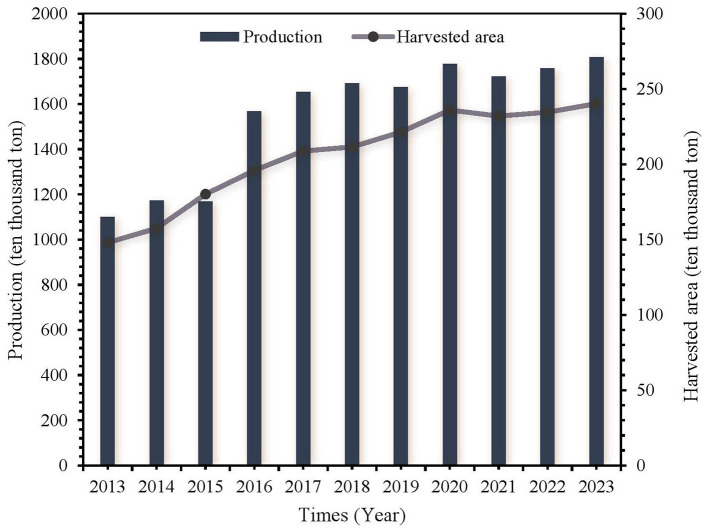
The harvest area and production of taro from 2013 to 2023, globally.

Taro (*Colocasia esculenta* L.) is an underground tuber of taro, which belongs to *Araceae* family. It is also known as hairy and fragrant taro. Taro's origins are associated with southern India and Southeast Asia, especially the Indomalayan region. It has been widely distributed to other regions, such as China and the Malay Peninsula, and now cultivated in tropical and temperate regions worldwide. Human migration and trade facilitated the spread of these to various parts of the world, significantly impacting human societies and their development (1). *C. esculenta* is a major agricultural staple food crop with more than 10 MTs produced annually around the globe (2). According to FAOSTAT, the global annual taro production reached 18.074 Mts in 2023, and the harvesting area of 2.402 Mha. The production has increased by 64.37% and harvesting area by 62.37% as compare to 2013 (Figures 1–3).

**Figure 4:**
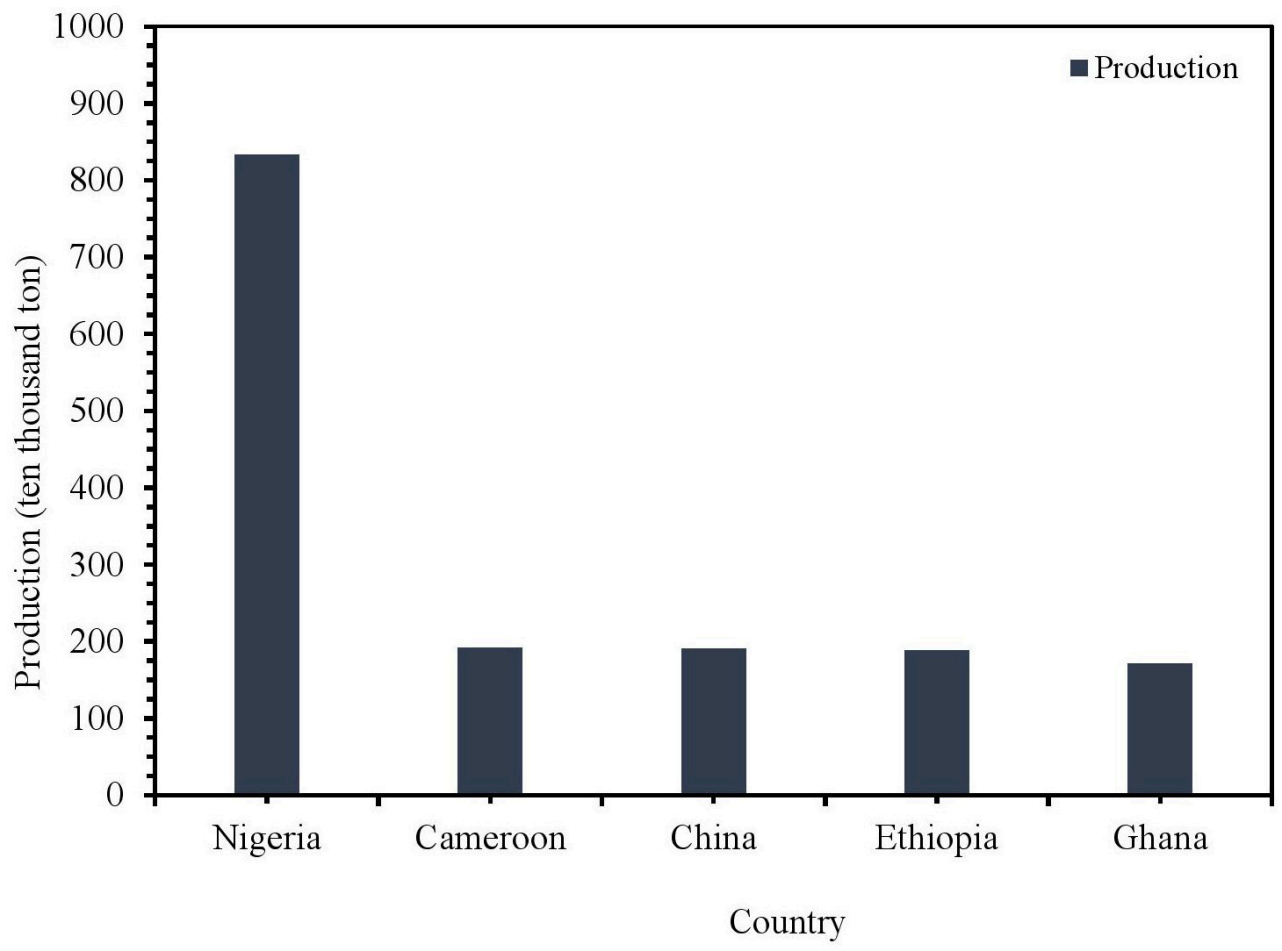
The production of taro from top five countries around the globe (2023).

This figure represents the scale of taro in global food production and highlights its importance as a human food requirement. Nigeria leads in taro production globally, followed by Ethiopia, China, Cameroon, and Ghana. Specifically, Nigeria accounts for 25% of the world's taro production (Figure 4). Africa leads global taro production, contributing nearly 14.78 Mts from 2.28 Mha, with an average production of 6.49 tons per hectare. In Ethiopia, taro plays a major role in the agricultural sector, with current production estimated at 1.69 Mts from 65,223 ha, yielding an impressive average of 25.92 tons per hectare. Ethiopia, as the second-largest producer of taro in Africa, following Nigeria. In Ghana, taro germplasm is found in farmers' fields and forested regions. However, this genetic resource is gradually disappearing due to the expansion of cereal crops, recurring water deficit, and deforestation. Taro has become a smart future food crop under the category of underutilized roots and tuber crops to combat chronic malnutrition and hidden hunger in Asia, especially in densely populated areas (3, 4). In Japan, taro was traditionally seen as a poor man's replacement for rice. It is often served as a side dish in various restaurants. It is a popular royal food in Cyprus, as well as a traditional food of Egypt's minority Coptic culture on New Year's Eve and it is a crop of high social status among Polynesian chiefdoms. People in Samoa claimed that Samoans are the folks from the sun, and that taro played a potential role in their ancestors' migration (5).

As a major taro producer, China has become the key taro industry globally. In 2023, taro production reached 1.948 Mts and harvesting area 99,600 Ha. Taro contains various proteins, vitamins, starch, and mineral elements (6). It is not only a standard raw food material, but also used as a medicine. It has a tremendous therapeutic effect on diseases, such as swelling, psoriasis, and burns. However, some challenges, such as relatively limited production scale and in-depth processing research, still limit the development of taro industries (7). In China, taro cultivation has a history of over 2,000 years. The ecological diversity has resulted in rich taro varieties. In China, the most considerable taro germplasm resources are available in the Wuhan Aquatic Vegetable Resource Garden, which had collected more than 400 germplasm resources by 2013 (8). Currently, there are more than 2,000 aquatic vegetable germplasm resources are preserved. The Wuhan Institute of Botany has been actively collecting these resources from various parts of China, including Guangxi regions. These collections are valuable for research and conservation efforts. In 2018, Jiangxi province established a strategic alliance for technological innovation in the high-quality taro industry, which promoted the technological progress of taro industry. By searching relevant literature, the China Geographical Indications Network found that 23 taro varieties are listed as geographical indication agricultural products (Table 1).

**Table 1:** List of taro varieties cultivated as regional basis in China.

**Registration year**	**Product name**	**Province**
2008	Jiangyong taro	Hunan
2010	Baimiao taro	Shandong
2010	Laiyang taro	Shandong
2011	Jianchang red taro	Jiangsu
2011	Fuding areca nut taro	Fujian
2011	Tufang areca nut taro	Fujian
2011	Denglong floury taro	Jiangxi
2012	Jingjiang taro	Jiangsu
2013	Yongding June red multi-clone taro	Fujian
2013	Yanshan red bud taro	Jiangxi
2014	Haimen fragrant taro	Jiangsu
2014	Tanbu areca nut taro	Guangdong
2015	Shiming taro	Fujian
2015	Lipu taro	Guangxi
2016	Wangqiao flower-bud taro	Jiangxi
2016	Wannian fragrant sandy taro	Jiangsu
2016	Taixing fragrant taro	Jiangsu
2016	Liuxu taro	Guangxi
2018	Yongkang divine taro	Zhejiang
2018	Yanglin goutou taro	Hubei
2019	Shagou taro	Shandong
2021	Xinmao taro	Jiangsu
2022	Anhe taro	Guangxi

**Figure 5:**
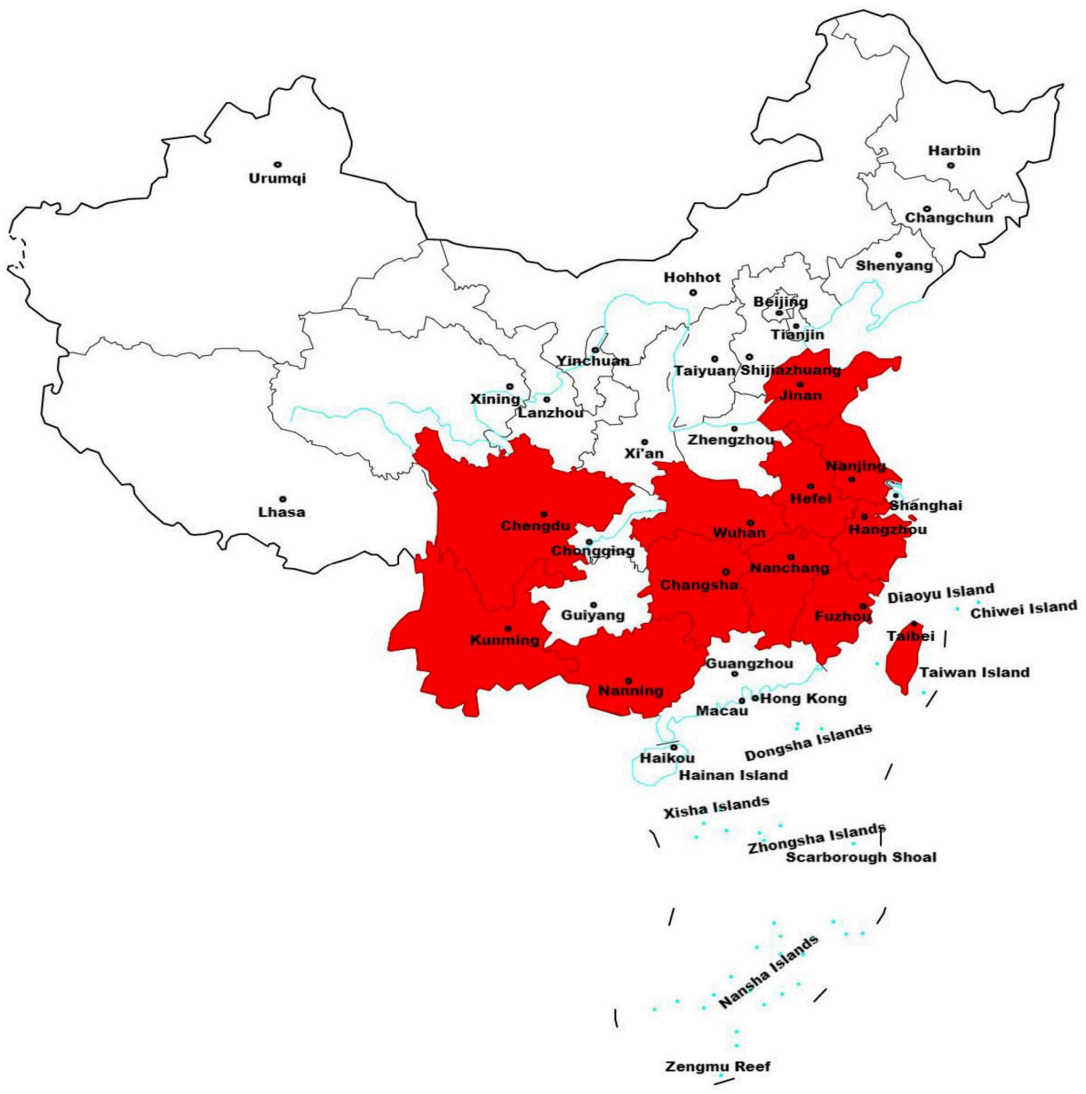
The major taro producing regions in China.

The major taro producing areas are the Guangxi Zhuang Autonomous Region, Guangdong, Fujian, Hunan, Shandong and Sichuan provinces, the Pearl River, Yangtze River and Huaihe River basins, and Taiwan, etc. (9) (Figure 5). The varieties planted in the main producing areas are shown in Table 2. At the same time, the popularization and application of taro preservation and storage technology, low-temperature vacuum frying, and vacuum freeze-drying application, the global taro processing industries have also developing rapidly, becoming more industrialized and mechanized. This article highlights the recent advancements in understanding taro's nutritional value, its health benefits, and potential for various applications, providing valuable insights for future taro processing research and development.

**Table 2:** Significant varieties of taro grown in major producing areas in China.

**Production unit**	**Mainly cultivated varieties**
Guangxi	Lipu taro, Hezhou fragrant taro, Gui taro 1, Gui taro 2, Rongshui fragrant taro, Liuwei fragrant taro, Beihai fragrant taro
Guangdong	Red handle taro, White taro, Hairy taro, Dog claw taro, Areca taro, Bamboo root taro, Purple taro, Red poplar water taro, Red bud taro, Tanbu areca taro
Hunan	Linwu taro, Jiangyong taro, Dog head taro, Chicken taro, White taro, White flour taro, Green stem taro
Shangdong	Laiyang solitary taro, Laiyang 8,520, Flower taro, Family king 1, White temple taro, Shichuan early born, Shagou taro, Lu taro 1
Jiangsu	Jingjiang taro, Jintan red taro, Taixing taro, Rugao Xiangtang taro, Jiangyan purple taro, Xinghualong taro, Taizhou longtou taro
Fujian	Bamboo shoot taro, Bamboo taro, White taro, White flour taro, Water taro, Ningde taro, Wine bottle taro, Long feet nine head taro, Red root without mother taro
Sichuan	Wulong leaf taro, String root taro, Red mouth taro, Lotus taro, Black stalk gun taro

## 2 Characterization and major nutrients of taro

### 2.1 Classification of taro

**Figure 6:**
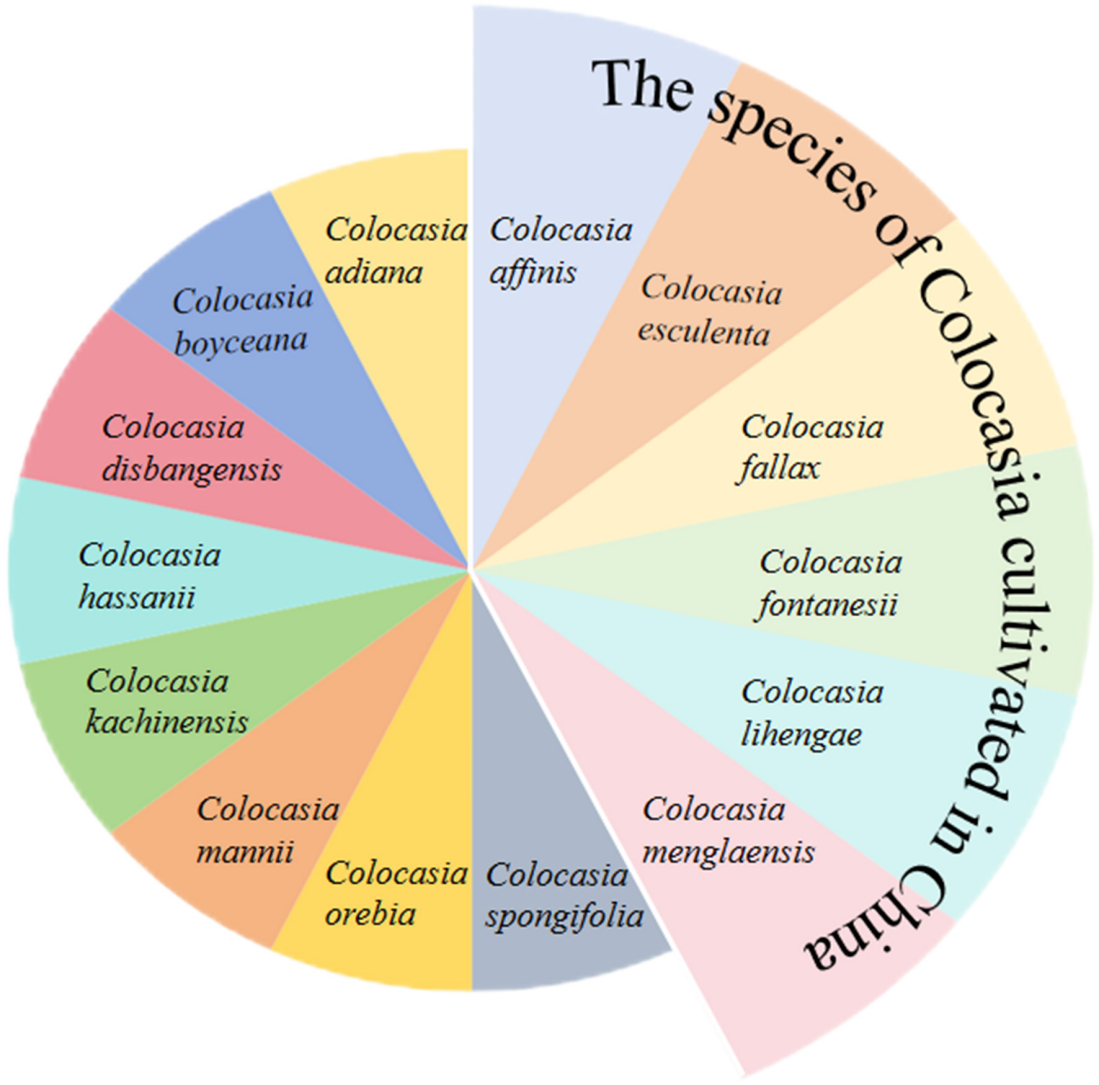
Classification of *Colocasia* species.

The genus *Colocasia*, belonging to the *Araceae* family, comprises ~20 species found in tropical and subtropical Asia, with more than 10,000 local varieties of taro (10). Six *Colocasia* species are available in China, and more than 400 taro varieties are preserved in the Wuhan Aquatic Vegetable Resource Garden (Figure 6). Taro plants are divided into long and short appendage variants (11). The short appendage of taro is divided into unicorm, multi-headed, and multi-seeded taro. According to cytological classification, it is divided into diploid and triploid (12). According to the classification of edible organs, Matthews and Nguyen (13) proposed the product organ petiole and corm variant first classified, then the corm variant was divided into three types, i.e., unicorm, multi-seeded, and multi-headed taro.

**Figure 7:**
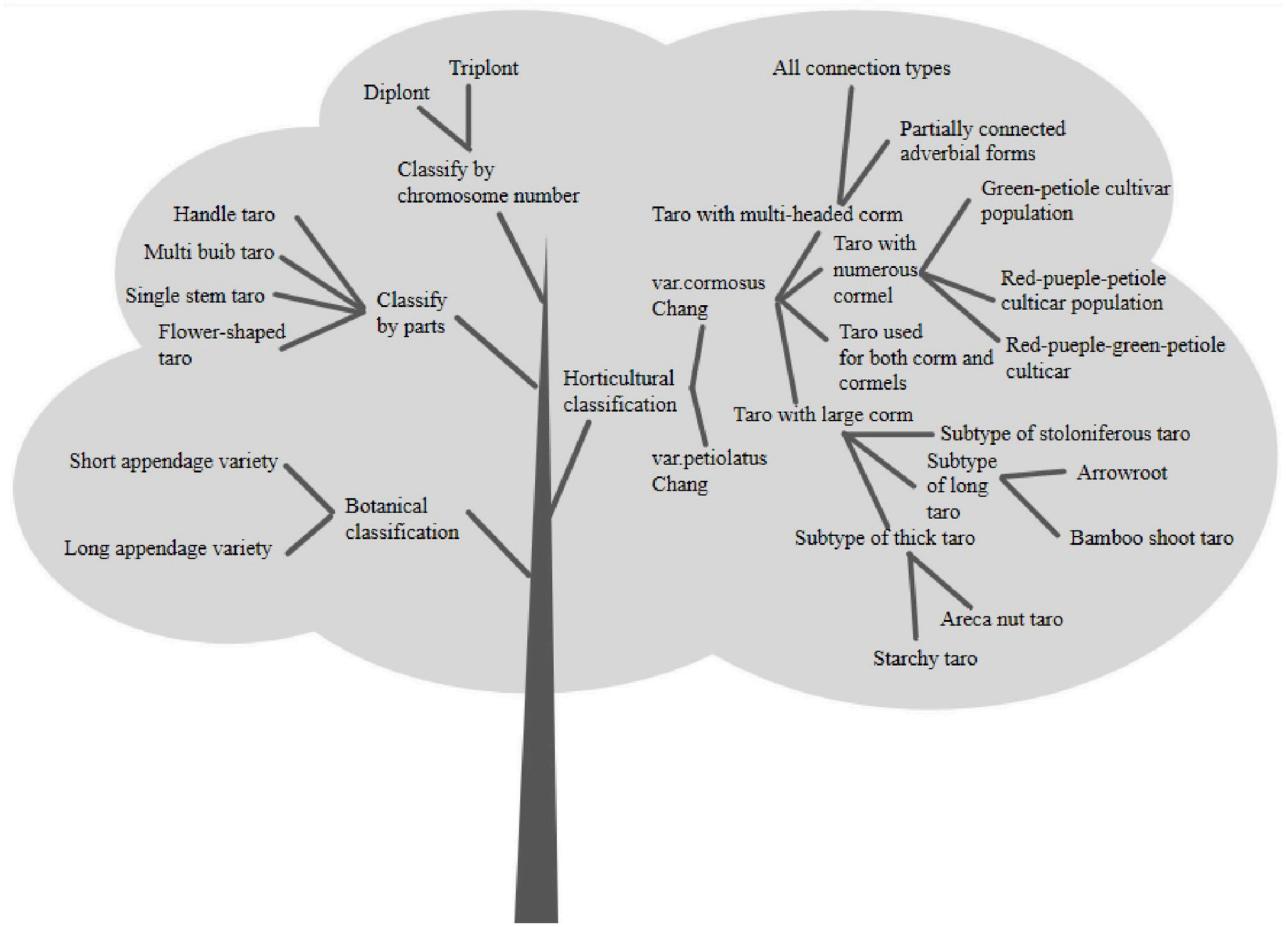
Classification of taro germplasm.

Each type can also be subdivided into subtypes according to the petiole's and taro's color (14). Xu et al. (15) classified into five types, such as, flower, single mother stem, multi-bulb (newly grown bulbs are larger than mother stems, newly grown bulbs smaller than mother stems), and stalk. Ebert and Waqainabete (16) classified bulb varieties into four categories, like big, big and seed taro, multi-seed, and multi-head taro, and found their storage tolerance was considerable big and seed taro < big taro < multi-seed < multi-head. Based on the available literature and relevant information, the different classification of taro is shown in Figure 7.

### 2.2 Major nutritional content of taro

**Figure 8:**
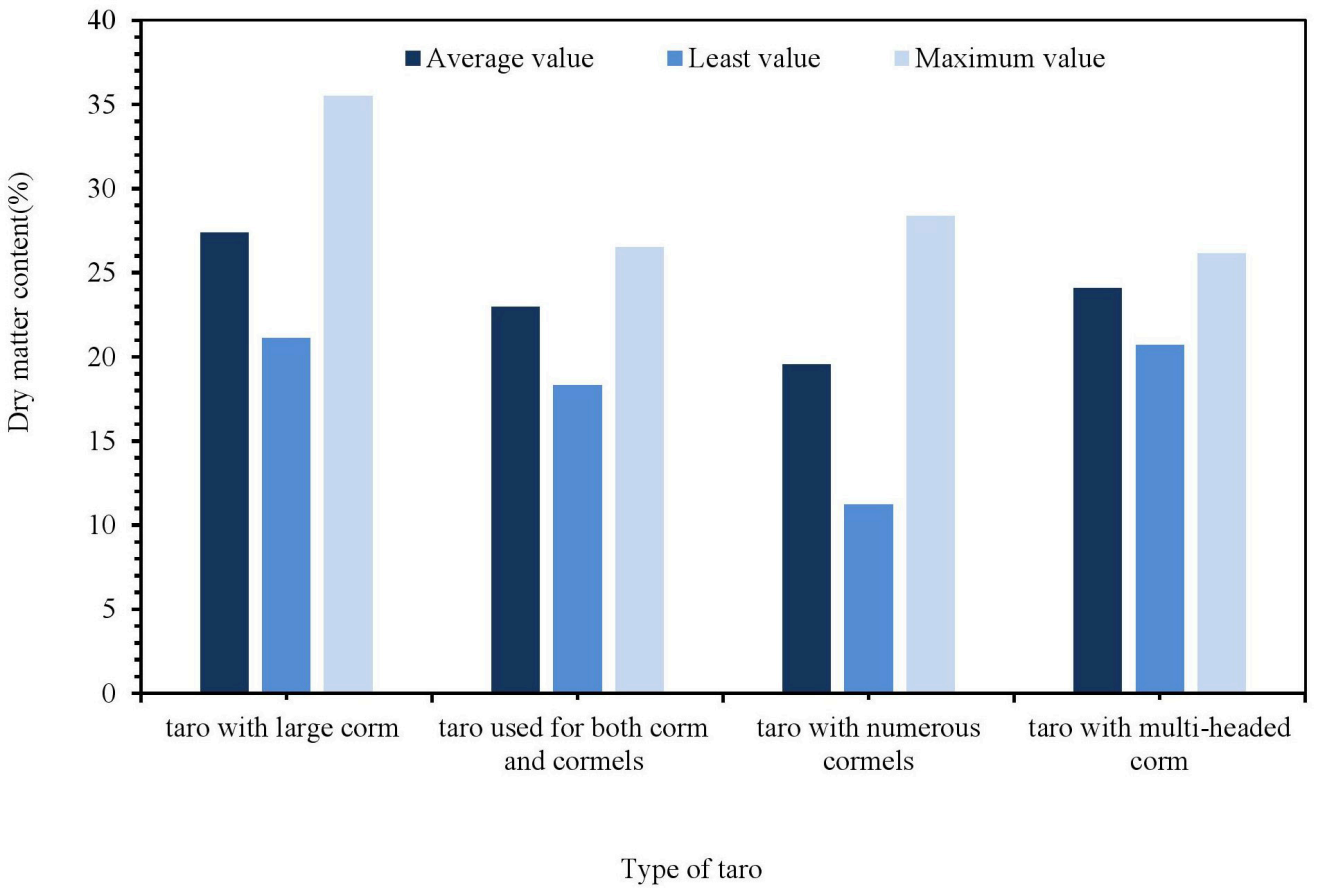
Impact of dry matter content with different types of taro.

Taro is indeed a nutrient-rich food source, boasting a combination of starch, dietary fiber, protein, soluble sugars, and minerals. These components contribute to energy supply and functional benefits (17). Different taro varieties and growing environments can lead to different nutrient contents (18). Zhu et al. (19) compared the material content of betel nut and multi-seed taro. The experimental results showed that the starch content of betel nut taro was about 24%. The multi-seed taro accounted for about 12%, and the anthocyanin content of betel nut taro was much higher than multi-seed taro. Huang et al. (20) analyzed the 206 taro species. They found that the dry matter content of taro was multi-seed < kuizi dual-purpose < multi-headed < kui taro (Figure 8). We et al. (21) determined the basic nutrients and contents of different varieties of taro under wet conditions during the quality comparison of different taro varieties (Table 3).

**Table 3:** The nutritional components of different varieties of taro (18).

**Variety of taro**	**Starch/g/100 g**	**Saccharose/g/100 g**	**Reducing sugar/g/100 g**	**Soluble protein/mg/100 g**	**Vitamin C/mg/100 g**	**Total polyphenol/mg/100 g**	**Moisture content (%)**
Longxiang	15.87 ± 0.73	0.70 ± 0.15	1.18 ± 0.13	943.21 ± 91.36	4.48 ± 0.26	39.67 ± 4.51	80.65 ± 0.32
Fenghua	16.07 ± 0.95	0.39 ± 0.07	0.80 ± 0.07	426.83 ± 54.65	0.56 ± 0.10	0.56 ± 0.10	82.94 ± 0.38
Betel-nut	21.80 ± 2.61	1.84 ± 0.42	1.17 ± 0.06	512.40 ± 18.02	2.29 ± 0.17	27.46 ± 2.35	61.33 ± 0.70
Lipu	16.46 ± 1.98	2.60 ± 0.24	0.68 ± 0.12	827.27 ± 55.74	3.65 ± 0.54	42.74 ± 2.51	66.64 ± 0.88
Xiangsha	30.25 ± 2.33	1.22 ± 0.07	0.81 ± 0.11	758.55 ± 91.36	1.79 ± 0.25	51.33 ± 3.63	66.51 ± 1.32

The nutritional components of taro are calculated based on wet weight.

### 2.3 Mucilage, starch, non-starch polysaccharides, and dietary fiber

Generally, taro mucilage has high carbohydrate content, primarily consisting of galactose, arabinose, glucose, and mannose (22). Taro contains a significant amount of mucilage (3%−19%), though the exact content varies depending on the extraction applications. This mucilage exhibits excellent functional properties, including high viscosity, water-holding capacity, oil-holding capacity, and antimicrobial activity. Due to these characteristics, taro mucilage holds great potential as a multifunctional ingredient, serving as a fat replacer, gel-forming agent, thickener, and emulsifier (6). Chemically, mucilage is a physiological byproduct of plant metabolism, primarily composed of polysaccharides, such as L-rhamnose, D-galactose, D-xylose, and L-arabinose. It also contains organic acids and trace amounts of protein (23). Unlike other root crops, taro stands out for its exceptionally high dietary fiber content. Studies show that raw taro corms contain around 13.5% fiber, while cooked corms retain about 3.21%. Additionally, taro flour has unique characteristics, such as small starch granules and high mucilage (gum) content, making it an excellent ingredient for alternative bread and noodle formulations. These properties allow taro-based products to serve as viable substitutes for traditional wheat or corn starch alternatives (24, 25).

Taro has the optimum starch content. Research results showed that the starch content in every 100 gm of taro can reach 70–80 gm (26). The particle size of starch is about 1–4 μm (27). The Zhejiang Academy of Agricultural Sciences, China, determined the starch content of Fenghua taro is 46.04% (28). The starch content is relatively low (3.29%) in taro leaves (29). Varietal differences, parts, origins, and processing strategies cause the difference in the measured content of taro starch. Taro starch dissolution and absorption rate are slow with low GI food. It is suitable as a substitute staple food for diabetic patients and helps to control postprandial blood sugar fluctuations. People usually use taro starch to replace rice and wheat to make low-calorie staple foods, such as taro vermicelli, taro porridge, etc., and mix it with grains and beans to make composite flour to improve nutritional balance. For example, taro wheat flour is used for baking bread and biscuits, etc.

Polysaccharides are macromolecular compounds composed of multiple monosaccharides. They are the basic components of organisms and physiological substances required for survival (30). Taro contains different compounds of polysaccharides, with the content in the corm being 9.0% and petiole 6.50% (29). Research scientists obtained different polysaccharides through different extraction methods, such as YT-1-a, TSP1, TSP2, CE-4a, TP-WS-NSP, Trao-4-l and Tc-WS-NSP-ICE, etc. (31). Mostly polysaccharides obtained through preliminary extraction are heteropolysaccharides, which need further purification for better research findings. Li (32) study separated YT-1-a in the study of taro polysaccharide extraction and obtained various monosaccharides, like xylose (30.79%), galactose (10.26%), rhamnose (17.77%), mannose (10.92%), glucose (10.20%), and galacturonic acid (7.03%). The non-starch polysaccharides in taro act as prebiotics, promoting the proliferation of beneficial bacteria, such as bifidobacteria and lactic acid bacteria, inhibiting pathogenic bacteria, such as *Escherichia coli*, and reducing the levels of intestinal inflammatory factors, i.e., TNF-α and IL-6. In the food processing industry, it is used as a functional additive in yogurt and beverages to enhance the prebiotic properties of the product.

Dietary fiber is called a “scavenger” and can promote intestinal peristalsis. The comparison of the nutritional components of nine aquatic vegetables in Wuhu city by Biswas et al. (33) found that taro had the highest dietary fiber content. The dietary fiber content in taro is four times more than cabbage. The fiber content in taro (3.10%) and taro leaves (2.80%) (34, 35). Therefore, taro can become a source of dietary fiber in the human diet. The resistant starch and soluble fiber in taro form a viscous gel, which reduces the gastric and intestinal absorption efficiency of glucose, soluble fiber increases stool volume, stimulates intestinal peristalsis, and acts as a prebiotic to promote the proliferation of bifidobacteria and lactic acid bacteria. Taro dietary fiber (especially soluble fiber) can occupy stomach space after absorbing water and swell, delaying gastric time and producing continuous feeling of fullness, which is suitable for people who want to reduce body weight and control calorie intake.

### 2.4 Protein and amino acids

A group of researchers have determined the chemical composition of different taro varieties and found that Ethiopian taro's protein content is 6.43% (36). The protein content of three local taro varieties in Taiwan is 1.75%−2.57% (37). Among them, G1 (mannose binding lectin) and G2 (trypsin inhibitor) proteins are essential for patients with food allergies (6). Previous research studies have shown that the taro protein can be hydrolyzed entirely to obtain seven essential amino acids for humans (38). Jiang et al. (39) determined the umami amino acid content of red bud taro protein was optimum, accounting for 40.43% of the total amino acids. Therefore, red bud taro is much more tender than ordinary food and rich in lysine. It is rich in lysine, which can make up for the lack of lysine in the body. Taro leaves also contain 26.84% protein (40), and the types of amino acids contained after hydrolysis.

### 2.5 Minerals, sterols, vitamins, and carotenoids

Taro contains a variety of trace elements required by the human body. Taro is also a good source of potassium, magnesium, and vitamins C and E. Luis-González et al. (41) applied absorption spectroscopy to determine the content of sodium, potassium, calcium, magnesium, iron, and other elements in the edible part of taro. The mineral contents are categorized as K, Ca, P, Fe, Mg, Na, Cu, and the Na/K values < 0.2. It is a low-sodium and high-potassium food. Low-sodium salt substitutes have been developed to help hypertensive patients control their diet. Christou et al. (42) determined the content of chemical compounds in taro stalks of different colors during different harvesting seasons. They found that the mineral content of green taro stalks reached optimum in July and August, respectively. The fluoride and saponin components in taro have antibacterial effects and can be extracted for food preservation technology in the near future.

Sterols are one of the beneficial active chemical substances in plants. The sterols currently in taro include stigmasterol, β-sitosterol, brassinosteroid, etc. Stigmasterol, a phytosterol found in plants, has demonstrated various beneficial biological activities and therapeutic potential in research. It exhibits cardioprotective, liver-protective, anti-inflammatory, and anticancer effects, and shows promise in treating conditions like arthritis, oxidation, and cholesterol-related issues. β-sitosterol can reduce low-density lipoprotein cholesterol, prevent prostate diseases, promote ulcer healing, and inhibit epidermal inflammation. Its promotion and application still require more research demonstrations. Brassinosteroids have a high affinity for plants and widely used. It is the sixth largest plant hormone, mainly used in agriculture, horticulture, and other related fields to promote plant growth and upregulate yield and quality (7).

The root and leaves are excellent sources of several vitamins. Key vitamins found in taro include vitamin E, C, niacin, and folic acid (31). The vitamin E in taro works synergistically with dietary fiber to protect cell membranes from oxidative damage and delay aging. At the same time, it can help to prevent arteriosclerosis by regulating lipid metabolism. Vitamin C can regulate different biochemical processes, such as synthesis of collagen (43), and acting as a cofactor of epigenetic enzymes during embryonic development (44). Since humans cannot produce ascorbic acid, the only source of vitamin C is from the diet (45). Niacin is the most physically and chemically stable vitamin, which promotes cholesterol metabolism and improves skin barrier function. The human body's daily requirement of niacin is 1.3 mg. Hundred g of taro contains 0.734 mg of niacin, meaning eating 200 gm of taro daily can meet the human body's needs (31). Folic acid is a water-soluble vitamin that comes from the natural and synthetic forms. It reduces neonatal neural tube defects, anemia, cardiovascular diseases, and other diseases (46).

Polyphenol compounds, found in a variety of plants, possess significant potential for medical applications due to their diverse biological activities. Researchers have proved through research experiments that polyphenols have a therapeutic effect on type 2 diabetes (47). Flavonoids are a secondary polyphenol compounds that can play a major role in different stages of viral infection and have multiple values for the human body, such as anti-inflammatory, antibacterial, anti-allergic, and prevention of cardiovascular diseases (48). In 100 grams of fresh taro, there are 28.04 mg of total flavonoids and 34.95 mg of total polyphenols. This indicates a relatively high concentration of these beneficial compounds in this edible root (29). The provided information states that 100 grams of fresh taro leaves contain 154.4 mg of total flavonoids and 250.23 mg of total polyphenols (6).

The composition and concentration of phytochemicals and antioxidants vary across different foods, consuming a diverse diet, especially colorful foods in each meal are essential. Key phytochemical groups that contribute to the total antioxidant capacity (TAC) of plant-based foods include polyphenols, carotenoids, and traditional antioxidant vitamins, such as vitamins C and E (49). Both total polyphenol and anthocyanin content serve as reliable indicators of antioxidant capacity, with studies demonstrating a strong correlation between antioxidant activity and polyphenol levels. Notably, certain flavonols (e.g., quercetin, kaempferol, myricetin) and carotenoids (e.g., lutein, lycopene, α-carotene, and β-carotene) exhibit potent antioxidant properties (50).

### 2.6 Alkaloids

Alkaloids are naturally occurring alkaline compounds and a key active component in Chinese herbal medicines. Studies have shown that taro leaves contain a significantly higher alkaloid content (11.02%) compared to taro residue (0.212%) (51). In terms of functional properties, Zubair et al. (26) demonstrated that taro stem alkaloids exhibit remarkable antioxidant activity, with a hydroxyl radical (–OH) scavenging capacity 10.9 times higher, an overall antioxidant capacity 3.5 times greater, and DPPH free radical inhibition 8.3 times stronger than fresh taro stem juice.

## 3 Efficacy of taro

### 3.1 Regulating blood sugar and blood lipids

Advance medical research shows that taro significantly regulates blood sugar (32). The fructose and glucose content in each 100 g of taro was found to be 0.63 and 0.55 g, respectively, and sucrose content was undetected (33). Compared with sucrose, fructose and glucose are monosaccharides and easily absorbed by the human body. Fructose is sweeter than glucose but lower glycemic index. Taro starch also contains specific proportion of resistant starch (52), which gives it a unique advantage in carbohydrate nutrition. Resistant starch is a special starch that is difficult to break down by digestive enzymes in the small intestine (53), thereby avoiding a rapid increase in blood sugar. It enhances satiety and reduces energy intake, which has positive significance for controlling weight and preventing obesity. Taro is an ideal food for diabetic patients looking to control their blood sugar. Added taro in the daily diet can meet the body's demand for carbohydrates, help control blood sugar to a certain extent, and reduce the adverse effects of blood sugar fluctuations in the human body. Research team found that taro is a low-sodium and high-potassium food. Potassium can also promote sodium excretion, reduce sodium and water retention, and reduce blood pressure. For patients with hypertension, increasing potassium intake can help to control blood pressure and reduce the cardiovascular disease risks.

### 3.2 Regulating intestinal flora

Taro contains more starch, including a special substance called resistant starch. Resistant starch can enter the large intestine and ferment under the action of intestinal microorganisms to produce short-chain fatty acids, such as acetic acid, propionic acid, and butyric acid (54). These short-chain fatty acids provide energy source for beneficial intestinal bacteria, promote the growth and reproduction of beneficial bacteria, maintain the balance of intestinal microecology, and regulate the secretion of intestinal hormones. Taro is rich in dietary fiber, which helps to promote intestinal peristalsis, improve the balance of intestinal flora, and possible reduce the risk of colorectal cancer. Plant polysaccharides exhibit a wide range of beneficial functions in health-related applications. They can act on the host's microbial flora and promote the proliferation and differentiation of beneficial microorganisms to prevent diseases, such as diabetes, enteritis, cancer, cognitive impairment, and obesity (55). Polysaccharides of taro have unique functions in regulating intestinal flora. Polysaccharides can specifically stimulate the growth of beneficial bacteria, such as bifidobacteria and lactic acid bacteria in the intestines, optimize the intestinal microecological environment, enhance the intestinal barrier function, reduce inflammation, and help the metabolic balance of human body. This provides a significant response for plant polysaccharides to maintain health by regulating microbial flora.

### 3.3 Anti-inflammatory

Taro contains different active substances, including flavonoids, saponins, phenolic compounds, etc. Studies demonstrated that saponins and alkaloids in taro possess anti-inflammatory properties (56). The dietary fiber in taro can work synergistically with mucin to form a strong protective film in the intestine, effectively reducing the release of pro-inflammatory factor TNF-α, thereby reducing the risk of intestinal inflammation. Anthocyanins in taro have antioxidant and anti-inflammatory effects (57). Japanese research teams have discovered through in-depth studies that the unique ceramide compound in taro can significantly reduce intestinal CRP levels, a key marker of inflammation. It offers a scientific foundation for understanding the dietary habits of Okinawa's long-lived population. They used taro as their staple food. This traditional diet may have relied on the anti-inflammatory effect of ceramide in taro to a certain extent, helping to maintain intestinal health and provide a solid foundation for longevity.

### 3.4 Antioxidant

During normal metabolism, the human body continuously produces free radicals. Excessive free radicals lead to the occurrence a variety of diseases. Antioxidant components, such as polyphenols and vitamin C in taro, can scavenge free radicals and reduce the inflammatory response caused by oxidative stress. Most plant polysaccharides also inhibit lipid peroxidation and scavenging free radicals (58). Talang et al. (29) proved through the study of antioxidant activity of taro polysaccharides that crude polysaccharides and purified polysaccharides have a strong ability to scavenge free radicals, and the concentration of polysaccharides is proportional to the ability to scavenge free radicals. The quercetin content in taro is higher (3–5 times) than ordinary vegetables. This antioxidant can effectively scavenge free radicals and inhibit the cyclooxygenase activity.

### 3.5 Antibacterial effects

Alkaloids, saponins, and tannins may inhibit microbial growth by destroying bacterial cell membranes, inhibiting bacterial enzyme activity, or interfering with DNA replication. Pereira et al. (59) also found that the taro lectin tarin has bactericidal activity after being purified by chromatography. Through macroporous resin experiments, Yakoubi et al. (60) found that alkaloids could effectively inhibit *Escherichia coli, Staphylococcus aureus*, and *Bacillus subtilis*. Taro also contains active ingredients, such as flavonoids and phenolic compounds, which work synergistically with polysaccharides and saponins to enhance the overall antibacterial effect of taro. Flavonoids have antioxidant and antibacterial effects, which can remove free radicals produced during bacterial metabolism, destroying bacterial DNA and protein structures, inhibiting bacterial growth. Phenolic compounds can interfere with bacterial metabolic activities by destroying the peptidoglycan wall of bacterial cells and permeabilizing the cytoplasmic membrane (61).

### 3.6 Anticancer activity

Cancer is becoming the leading cause of death in humans. Many studies have shown that relevant ingredients can be extracted from fruits, vegetables and other food materials to develop cancer drugs, including breast cancer, colon cancer, etc. (62, 63). Taro contains various bioactive molecules that can reduce the probability of cancer and related diseases, while enhancing metabolism and immune response. Polyphenols, carotenoids and flavonoids in taro are active compounds with potential anticancer properties (64–66). The polyphenols have therapeutic effects on breast, liver, and colon cancer, inhibiting tumor growth through multi-dimensional effects, such as anti-oxidation, pro-apoptosis, anti-proliferation, and immunomodulation. Its molecular mechanism involves key signaling pathways and epigenetic regulation. Sui et al. (65) found through extraction analysis that consuming an appropriate amount of carotenoids can reduce cancer risk. The polysaccharide compounds in taro have been recognized to inhibit a variety of tumor cells and low side effects on normal cells, and have become a potential resource for antitumor drugs.

Gastric cancer is one of the five most common cancers. High-fiber diet has been confirmed by various research demonstrations to associate with reduced risk of digestive system cancer. Esposito et al. (67) found that the antitumor activity of taro using an *in vitro* model of gastric adenocarcinoma, isolated 10 compounds, and proved that taro is effective in resisting gastric cancer.

### 3.7 Immune regulation

As a traditional food ingredient, taro has gradually attracted attention for its potential immunoregulatory effect. Taro is rich in dietary fiber, especially soluble fiber, which can be used as a prebiotic to promote the proliferation of beneficial intestinal bacteria. Healthy intestinal flora can regulate the activity of immune cells. Substances, such as butyrate, produced by intestinal flora fermentation of fiber, may enhance intestinal barrier function and reduce inflammatory response. The most significant biological activity of plant polysaccharides is their ability to modulate immune responses. Taro polysaccharides enhance immunity by modulating the proportion and differentiation of immune cells. At the same time, they can also stimulate macrophages to secrete antigens to eliminate pathogens and improve the body's ability to resist infection (15). Zhang et al. (68) obtained a non-starch neutral polysaccharide TPS from taro. Through mouse experiments, it was proven that TPS can enhance cellular and humoral immunity *in vivo*. In addition, taro contains glycoproteins, which can enhance the immune system. Taro is an alkaline food that can neutralize acidic substances. It prevents and protects excessive gastric acid and beautifies the skin.

### 3.8 Preventing obesity and other effects

Taro contains some water-soluble dietary fiber, which expands in volume after absorbing water, reducing food intake, and avoiding excess energy. At the same time, dietary fiber can combine with fatty acids; preventing fatty acids from being absorbed by the human body and protecting excess absorbed fat and obesity. Research has found that resistant starch in taro can also help to achieve weight control by enhancing satiety, reducing energy intake, and accelerating fat decomposition. Willis et al. (69) found that foods with high resistant starch content can stimulate satiety and prolong digestion time more than low-fiber foods, thus achieving weight loss effect.

**Figure 9:**
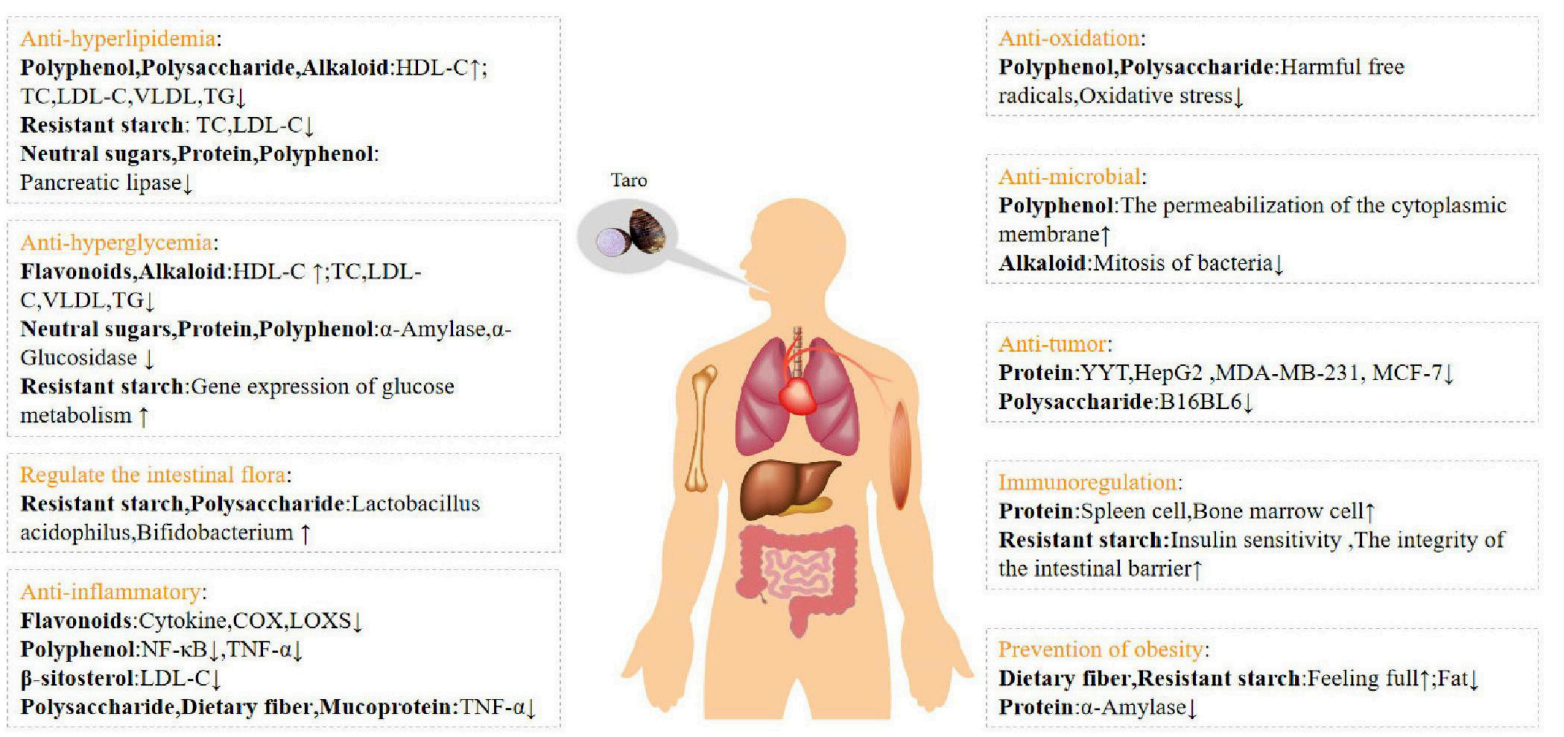
Nutritional and physiological effects of taro (*Colocasia esculenta*) on human body.

Increasing dietary fiber intake by eating taro can enhance the oral muscles and teeth chewing, preventing tooth loss. Taro has a high fluoride content, which can play a significant role in cleaning teeth and preventing caries. The dietary fiber in taro can combine with cholesterol to promote the circulation of bile in the body and effectively prevent gallstones; resistant starch controls insulin secretion, reduces cholesterol synthesis and prevent gallstones. Relevant animal experiments have proved that adding resistant starch to food can promote the nutrition's absorption efficiency, such as vitamins and minerals (Figure 9).

## 4 Major products and processing technologies

### 4.1 Main processed products of taro

**Figure 10:**
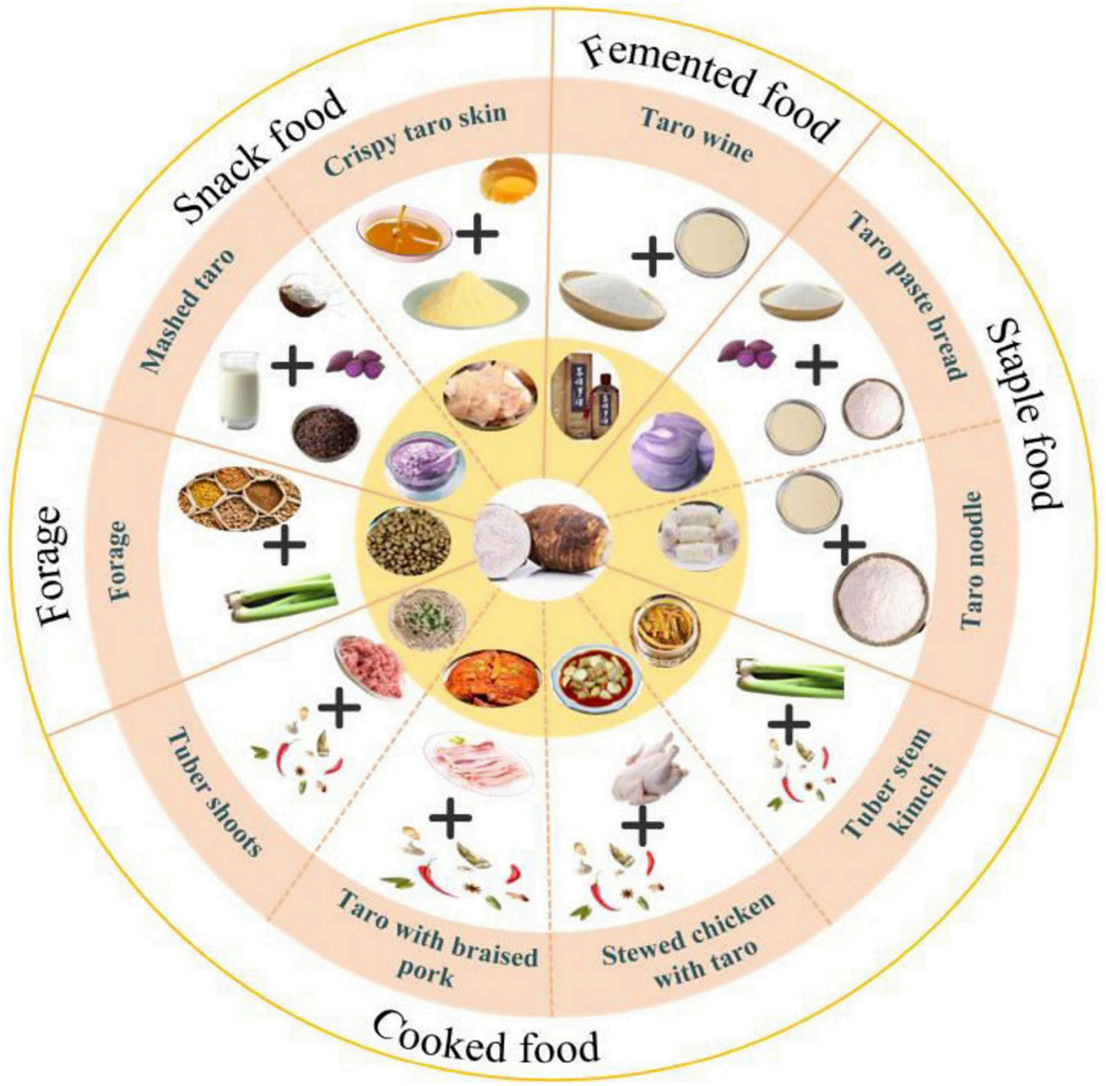
Processing diagram of some taro products.

Due to its unique taste and nutritional value, taro is widely processed in various products, covering the fields of food, industrial raw materials, etc. (Figure 10).

#### 4.1.1 Snacks, fermented, and condiment foods

Snack foods are the main products derived from taro deep processing. In the food industry, taro flour is often used to make cakes, potato chips, biscuits, and other products, such as Thai taro flavored coconut rolls, taro tamarind crisps, and American Lay's potato chips. Mixing wheat flour and taro flour in different proportions can make cakes with different flavors (70). The taro flour can replace 30% of wheat flour, with the effect of taro replacing wheat flour on biscuits (71). Qu et al. (72) found that by controlling variables, the dragon fragrant taro strips were crispy and had a strong taro flavor when fried for 115 s (160 °C). Taro paste is homogenized with sufficient water to form the required paste, and then processed into different flavors according to different materials (73).

Currently, the foods fermented with taro, i.e., wine (74), yogurt (75), and beverages (76). Sapal is a wine made from taro fermented in Papua New Guinea. Fresh taro is steamed and fermented at room temperature in a ratio of taro and coconut milk (5:1). They developed some fermented foods rich in taro, which have multiple functions, such as softening blood vessels and enhancing digestion efficiency (76).

Vinegar brewed from taro not only has the effect of fruit and vegetable fermentation, but also has the flavor of traditional vinegar. Takeuchi and Nagashima (77) produced taro vinegar through acetic acid fermentation of taro alcohol fermentation broth, resulting in a pleasant flavor. Taro sauce is rich in nutrition. Li et al. (78) used multiple strains to make koji and mixed fermentation to produce taro sauce. Sit et al. (79) compared the effect of adding the same amount of taro and corn starch to tomato sauce and found that tomato sauce containing taro starch tasted better. Related products, such as coconut taro paste sauce, taro fermented tofu, etc., have sufficient market value.

### 4.2 Other byproducts

**Figure 11:**
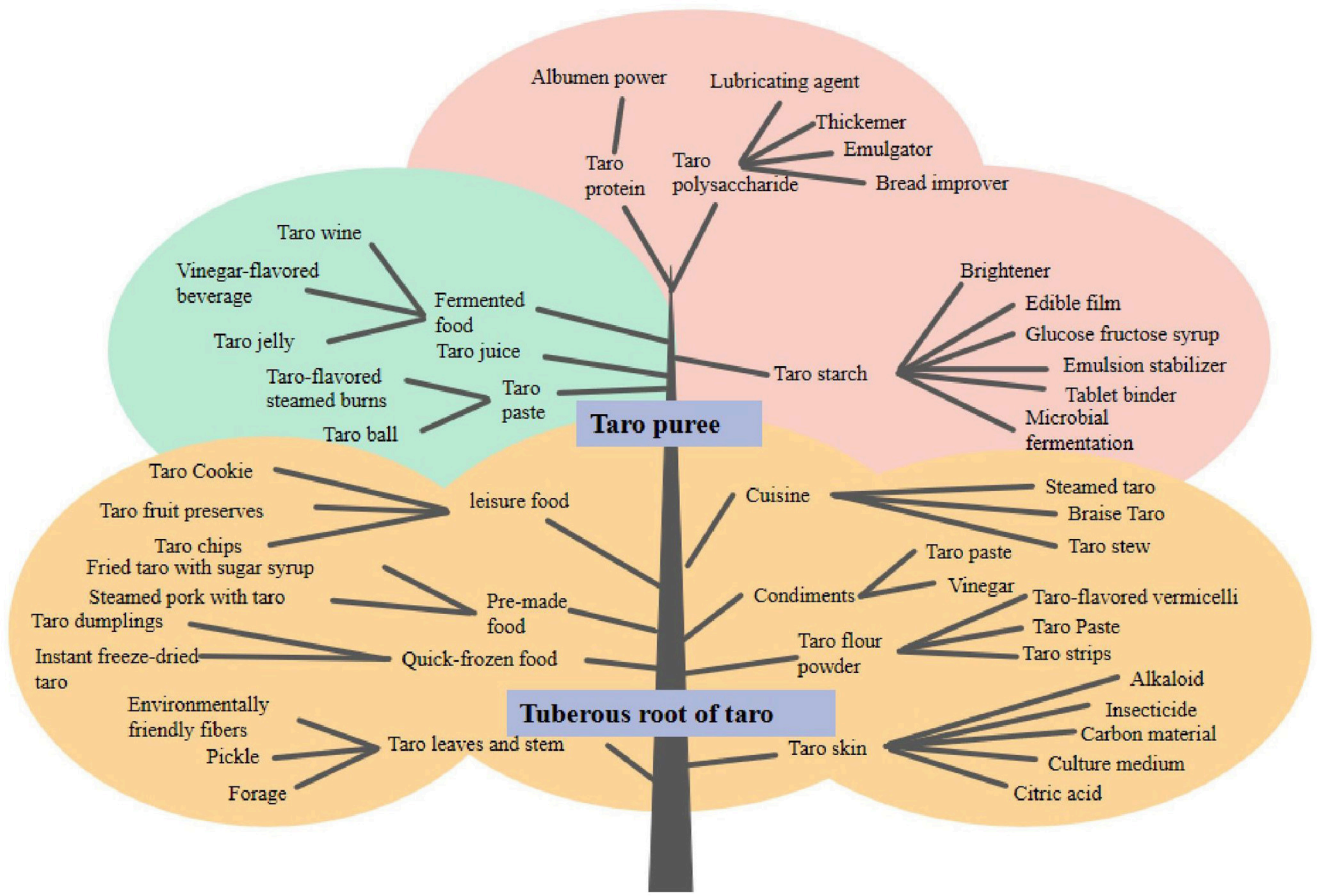
Taro processing flowchart.

In order to avoid waste of resources, Hu et al. (80) removed the astringency of taro petioles by blanching and low-salt fermentation. Taro petioles make side dishes by pickling (81). By products, such as taro peels produced during the process, are also used in different industries as environmentally friendly fibers (82), carbon materials (83), natural pesticides (84), biodegradable films (85), and feeds (9). Taro processed products are gradually gaining attention in the international market, which provides new opportunities for the further development of the taro industry (Figure 11).

## 5 Research and development on storage and processing technologies

Research and development on taro processing technologies are significant factors in the development of taro industry. It can not only extend the shelf life of taro and enhance its added value but also meet the requirements of different consumers and expand the market value.

### 5.1 Storage and preservation technology

Taro has a high starch content and resistant to storage. Traditional preservation approaches mainly include burial and cellar storage, and the shelf life is generally 3 months (86). The effects of moisture and packaging materials on taro storage and the shelf life of fresh taro at high temperature are about 2 weeks. The shelf life of dehydrated taro stored in polyethene bags is 38.5 weeks, and the shelf life of taro can be further extended by storing it in aluminum foil laminated bags with low water activity (87).

### 5.2 Compound extraction technology

There are different methods for extracting taro polysaccharides, mainly water extraction, microwave-assisted extraction, enzyme-assisted extraction, pulsed electric field-assisted extraction, and ultrasonic-assisted extraction (29). The primary methods for extracting globulin include Osborne fractionation, reverse micelle extraction, and ultrasonic-assisted extraction (88). Due to the similarity between proteins and polysaccharides, the proteins in taro also dissolved when extracting polysaccharides, so they need to be purified. Currently, trichloroacetic acid, hydrochloric acid, enzymatic, and other methods are commonly used to remove proteins. The extraction methods of taro starch include alkaline extraction, water precipitation, ammonia precipitation, double enzyme, and enzyme applications (89). The alkaline extraction method uses NaOH, which is highly corrosive, and the water precipitation method has low extraction rate. These two methods are not commonly applied. Compared with the water precipitation method, the ammonia precipitation application reduces the sedimentation time. The starch recovery rate of double enzyme method using xylanase and neutral protease can reach 78.77% (90). By optimizing the enzymatic extraction process, researchers found that under the conditions of pH 10.0 and enzymatic hydrolysis temperature 41 °C, the extraction rate of taro starch reached 88.92% after 137 min of enzymatic hydrolysis (89).

Low eutectic solvent is a new type of environmentally friendly solvent and a good substitute for traditional biomass solvent. It has the advantages of high thermal stability, simple synthesis, low toxicity, and recyclability (91). By repeatedly recycling the DES solvent, production costs were reduced. Most DES materials come from the industrial waste, which promotes waste utilization.

Supercritical fluid extraction technology uses fluid in an environment above the critical temperature and pressure as an extractant to extract specific components from non-liquids, leaving pure taro natural flavor extracts without significant changes in pH value, color, etc. (92). Supercritical fluid technology can also efficiently collect phenols, flavonoids, carotenoids, and other compounds in taro, and develop products with antioxidant, immunomodulatory, and other functional effects.

### 5.3 Dehydration technology

Nowadays, the method for dehydrating fruits and vegetables include freeze, vacuum, spray, low-temperature, and convection drying (93). Freeze-dried fruits and vegetables have the best quality and highest nutritional value (94), but the equipment used is expensive and consumes much energy. Spray drying of taro can produce taro powder, which has the advantage of simple operation, but the utilization rate of thermal energy is not high (95). Vacuum drying of fruits and vegetables retains good nutritional content and color, and suitable for products with high dry matter content (96). Compared with traditional hot air and vacuum drying, microwave vacuum drying technology has a shorter drying time, lower energy consumption, works at low temperatures, inhibits taro's oxidation rate, and improves taro's taste and color. Vacuum-dried different varieties of taro were microwaved, and the quality changes were compared. They found that the taste, flavor and color of Longxiang taro were the best (21).

### 5.4 Extrusion puffing technology

Extrusion puffing technology is a non-frying processing technology with low cost, large production volume, and limited loss of raw material nutrients. It is divided into direct and indirect puffing (97). It is mainly used in cereal breakfasts, snack foods, etc., enriching the variety of food. Applying extrusion puffing technology to grain processing can improve the product's taste. By adding purple potato powder to puffed powder, it was found that the flour yield can be increased and the shelf life of food can be extended. Rodríguez-Miranda et al. (98) used concentrated taro, black glutinous rice paste and corn starch as raw materials. They applied a combination of twin-screw extrusion and traditional frying to process taro crisps, providing a new idea for the deep processing of taro.

### 5.5 Vacuum low-temperature frying technology

Vacuum low-temperature frying technology is widely applied in producing fruit and vegetable crisps. It refers to the processing technology of low-temperature frying (95 °C) under vacuum, rapid dehydration and drying, and reducing the temperature effect on food nutrients. When applied to the production of taro crisps, the resulting product has low water and oil content, and the vitamins and minerals in taro are retained. Vacuum low-temperature frying technology has initially formed large-scale and industrialized production worldwide. With the continuous advancement of this technology, vacuum low-temperature fried foods will become a development trend. The research on taro processing technology continues to deepen, providing strong support for developing the taro industry. By continuously optimizing processing technology and technical parameters and developing advance processed products, the added value of taro can be improved, and market demand and sustainable development of taro industries can be promoted.

## 6 Development value of taro

The taro industry plays a vital role in multiple sectors, including the economy, food production, and medicine. A prime example of its success is Lipu taro, which stands as a highly representative case.

### 6.1 Economic value

Lipu city, Guangxi, China, has become an ideal place for taro cultivation due to its unique natural conditions. The local taro cultivation has a long history and better experience. In recent years, with the continuous growth of market demand for Lipu taro, the scale of cultivation has continued to expand. Lipu taro enjoys a high reputation in the national and international markets for its excellent quality and unique taste, and its brand value continues to improve.

The industrial chain is complete, and the scale of the industry is growing steadily. The annual processing volume of taro in Hezhou and Guilin, Guangxi, has reached 342,000 tons, and the processing output value reached 3.43 billion yuan. There are 56 typical processing enterprises, and more than 10 enterprises with an annual output value of more than 100 million yuan, involving various processed products, such as taro slices, wine, and rice noodles (28). In 2024, Guangxi established a complete industrial chain pilot, forming a complete industrial chain of variety protection-planting-circulation-storage-processing, and improving the ecological industrial chain of Guangxi taro. The development and growth of these enterprises not only developed more tax revenue for the local area but also promoted the coordinated development of upstream and downstream industries, formed a complete industrial ecosystem, and effectively promoted the prosperity of the local economy.

Brand effect aggregation, at the Lipu Taro Public Brand Release Conference and Taro Industry Summit Forum in Beijing, China in 2023. Fifty-eight thousand tons of lipu taro were sold, with a total amount of 776 million yuan. The brand effect of lipu taro has also injected strong impetus into the local economic development, increasing the market price of taro, attracting the attention of various companies and investors, and promoting the scale and industrialization of the taro industry. Different companies have entered the field of taro processing and developed a series of high-value-added products, increasing their profit margin.

As a speciality agricultural product, taro has a growing market demand. Its unique taste and nutritional values are favored by consumers, making it highly competitive in the market, and its price relatively stable and high. Taro has a significant yield potential. Scientific planting management can achieve higher yield, thus bringing considerable economic benefits to farmers. The labor-intensive nature of taro cultivation and processing provides different employment opportunities for the rural population and promotes the rural socio-economic development.

### 6.2 Edible value

Taro is a nutritionally balanced and adaptable food that combines the satiety of staple food with the health properties of vegetables. Its edible value can be divided into the following aspects.

#### 6.2.1 Direct consumption

As one of the world's oldest crop, taro became a staple food for humans much earlier than rice. Taro was used as a staple food in the Spring and Autumn period. Taro production peaked during the Tang Dynasty, when taro had the same status as millet in northern China. After the Southern Song Dynasty, the planting area of taro was reduced due to changes in crop rotation, but it still played an irreplaceable role as a food substitute. The low-fat and high-fiber characteristics of taro are suitable for modern dietary needs, and the starch particles are small, making it suitable for children and patients (99). Taro has a soft, glutinous and smooth texture, and can be eaten in different ways, including steaming, boiling, stir, and deep-frying. Famous dishes made with taro include braised taro, candied taro, taro pork, etc., as well as snacks, such as taro paste and taro milk. Lipu taro has become a geographical indication agricultural product with good color, fragrance, and taste, influenced by the environment and climate. Lipu taro is large, weighing 1 kg each, with soft and glutinous flesh and rich aroma. It is rumored that Heshen once presented lipu taro to the emperor, which became a must-have ingredient in the palace. In 2008, lipu taro became the designated taro for the Beijing Olympics in 2015, a national geographical indication agricultural product. It was successfully selected into the Chinese Agricultural Brand Catalog in 2019.

#### 6.2.2 Processed products

Taro has shown great value in the field of food processing. Its rich nutrients and unique taste provide a broad space for developing diversified processed products. The processing of lipu taro has transitioned from primary to intensive processing, involving multiple industries, such as snacks, fermented foods, and pre-prepared dishes.

### 6.3 Medicinal value

Taro products have an important position in the health industry. Their role in promoting the development of the health industry and meeting consumers' health needs is reflected in various aspects. Advanced medical research has demonstrated that taro's active compounds possess multiple health benefits, including anticancer properties, as well as the ability to lower blood lipids and blood sugar (100). Lipu taro is rich in nutritional value, containing abundant protein, anthocyanins, dietary fiber, vitamins, and trace elements, such as iron and zinc. It is known as the “best taro.” Dietary fiber helps to promote intestinal peristalsis, prevent constipation, and reduce the risk of cardiovascular disease. Vitamins and minerals participate in metabolism and physiological regulation, and maintain normal human body functions.

#### 6.3.1 Health care products field

The application of taro also provides new opportunities for the innovative development of the health industry. Taro contains special active substances, such as mucin and polyphenol antioxidant ingredients. These active substances have been proven to be effective in medical uses. For example, the expansion of taro polysaccharides can be added to orally disintegrating tablets to accelerate the dissolution of functional ingredients and quickly exert their effects (101). Sloan et al. (87) proved through enzyme inhibition studies on glycosidase that taro globulin has the potential to regulate blood sugar and high inhibitory activity on amylase.

The health value of taro also brings new opportunities and challenges to the development of health industries. It has prompted food companies to increase their research, development, and innovation efforts on taro-related products and continuously produce more healthy foods that meet consumer needs. It has also promoted the improvement of taro varieties and the improvement of planting techniques in the agricultural field to meet the market demand for high-quality taro.

### 6.4 Industrial value

Taro extracts also hold significant industrial value. Rich in polyphenols with antioxidant properties, they are used as natural preservatives in food and as key ingredients in health product development. Taro extracts can used in the cosmetics industry to produce skin care products and cosmetics with moisturizing and antioxidant effects. In the pharmaceutical industry, some components in taro have specific medicinal value and used to develop pharmaceutical or health products for the auxiliary treatment of diseases.

#### 6.4.1 Industrial raw materials

Lipu taro is rich in furans (dihydroagarwood furan), esters (methyl acetate), and olefins (cis- and trans-2,4-hexadiene) compared to other taro varieties (102). Furan is an oxygen-containing five-membered heterocyclic compound with aromatic ring properties. It can undergo electrophilic substitution reactions, such as halogenation, sulfonation, and nitration. It can be used in solvents to promote the reaction. Furan can react with various compounds to form hydrogen bonds, so it is also widely used in the biological field, such as DNA extraction, electrophoresis, PCR reaction, chemical raw materials, etc. Esters can be used as ingredients in flavors and fragrance products, providing fragrance in food, cosmetics, and detergents. It is used as a plasticizer to improve the durability and flexibility of plastics. Olefins are important compounds with industrial value. They are widely used in producing plastics, rubber, fine chemicals, and other fields. Olefins and esters can also be used as biofuels.

#### 6.4.2 Waste utilization

Taro processing waste has a specific utilization value. Taro peel and residue are rich in dietary fiber, which can be extracted and processed into dietary fiber powder for food fortification or feed additive. After the wastewater generated during taro processing, the organic matter can be extracted to produce biofertilizer, realizing the recycling of resources. The peel produced by taro processing can feed pigs, poultry, and other animals. Taro petioles are rich in crude protein and rich protein feed resource. After soaking, boiling, draining, and dehydrating, taro is fed to broilers in proportion. It was found that the daily weight gain of broilers enhances (103), when taro petioles were added with whey and molasses and ensiled for 8 days and fed to pigs instead of corn, the size of the pigs' large intestine and pancreas decreased, while the mass of their stomach increased (104).

### 6.5 Social value

Taro has important value in the economic field and plays a multi-faceted positive role at the socioeconomic level.

#### 6.5.1 Land protection

Taro cultivation is relatively adaptable, low requirements for soil and climate, and can grow on some relatively poor land. This makes taro cultivation of sustainable agricultural practice, contributing to land utilization and protection.

### 6.6 Cultural value

*Taro* is one *of* the most ancient cultivated crops *in China*. In some areas, taro is not only part of the daily diet but also carries specific cultural significance. Lipu city has held eight consecutive Lipu Taro Cultural Festivals in 2024, showing the developed agricultural economy and prosperous private economy of Lipu city, further enhancing the popularity and market competitiveness of lipu taro. As a benchmark for China's regional public brands of agricultural products, the brand value assessment of lipu taro has reached 2.626 billion yuan in 2024. With “fragrant and sticky” as its core value, lipu taro has extracted the brand slogan of “a mouthful of fragrant and sticky food for 300 years,” which has been disseminated through CCTV and other media, further strengthening its cultural symbol attributes. Taro is an important agricultural product and plays a significant role in the social economy, cultural heritage, environmental protection, and rural revitalization for better future.

## 7 Significance, conclusion, future research perspectives, and challenges

Taro has emerged as an affordable and nutrient-rich staple food, renowned for its significant nutritional, medicinal, and therapeutic benefits. With its potential to enhance food security, taro could serve as a vital crop, particularly in developing nations. It contains a high concentration of mucilage, widely recognized for its diverse industrial uses as well as its exceptional therapeutic and functional benefits. Additionally, it is rich in various carbohydrates and proteins, which significantly enhance the functional properties of food products. Taro starch offers significant health benefits, including blood sugar regulation, digestive health support, and rich supply of essential minerals and antioxidants. These qualities highlight its importance as a functional food ingredient. Additionally, its ability to enhance the nutritional value of food products along with its favorable physicochemical properties makes taro starch a versatile and valuable addition to food formulations.

However, research on taro storage and long-term quality preservation remains limited, and the lack of suitable industrial-scale processing equipment poses a significant challenge. Furthermore, greater efforts are needed to expand the global market for taro-based products, explore their potential applications, enhance processing conditions, and minimize post-harvest losses. Additional research and investment are essential to fully harness taro's potential in bolstering food security and driving economic growth, especially in developing countries. On the technical level, taro processing technology has made inevitable progress; more experimental research is required in the study of the physical and chemical properties of taro. In the process of taro starch extraction, the current extraction methods have some challenges, such as low efficiency and need to improve the purity of starch. It is necessary to develop more efficient and enviro-friendly extraction approaches. In terms of product development, various taro processed products have been developed, and the added values of the products are not optimum, and initial processing is still the main focus (105).

In terms of functional food development, further studying the nutritional components and physiological activity of taro and developing more products that meet the requirements of specific groups of people is still necessary. In terms of market, awareness and brand influence of taro products need to be improved. Nowadays, importing countries have listed some of China's exported food as unsafe food because the international standards are much higher than China's current standards (106). The number of domestic taro exporting countries will reduce to 30 between 2018 and 2023 (107), with exports mainly concentrated in Japan and the United States (108). The international market requirements have led Chinese taro companies to invest more funds and energy, reducing the export scale and revenue of Chinese taro-related products.

However, taro industry is also facing risks, such as fierce market competition, price fluctuations, and fruit and vegetable logistics and transportation. Need to strengthen technology and widely apply enviro-friendly and efficient advanced technologies to extract and develop taro's functional ingredients, promote mutual recognition of international food standards, and build cold chain logistics centers overseas to reduce export losses. In the era of health and precision nutrition, pay serious attention to healthy diet, the precise cognition and personalized demand for food nutrients are becoming more and more prominent, which pointout a new direction for the research and development of taro functional foods. In the near future, with people's nutrition and health, synthetic biology, and artificial intelligence as the two wings, a new platform for cooperation and sharing of time, space, information and resources will be built. In the field of smart future food, 3D printed food, simulated alternative food, plant-based food, intestinal health, and functional food.
